# A Comparative Study to Evaluate the Effect of Negative Pressure Wound Therapy and 3% Hypertonic Saline Dressings in the Management of Diabetic Foot Ulcers

**DOI:** 10.7759/cureus.111871

**Published:** 2026-07-01

**Authors:** Manjesh K A, Ashwini Dutt, Nagarjun Nelluri, Sreedhar Rao Kota, Kishore Abuji, Ankith Narra, Narender Mudigonda, Diya Venkata Sai Dheeraj, Eesha Agarwal

**Affiliations:** 1 General Surgery, Employees' State Insurance Corporation (ESIC) Medical College and Hospital, Hyderabad, IND; 2 Vascular Surgery, Sree Chitra Thirunal Institute for Medical Sciences and Technology, Hyderabad, IND; 3 General Surgery, Employees' State Insurance Corporation (ESIC) Medical College and Hospital, Hyderbad, IND

**Keywords:** 3% hypertonic saline, diabetic foot ulcers (dfu), hypertonic saline in diabetic foot ulcer, major limb amputation, management of diabetic foot, negative pressure wound therapy (npwt), vacuum-assisted closure (vac), vac vs 3% ns

## Abstract

Objective: Diabetic foot ulcers are a major cause of morbidity, prolonged hospitalization, and lower-extremity amputation among patients with diabetes mellitus. Negative pressure wound therapy (NPWT) has emerged as an advanced wound management modality with potential benefits over conventional dressings. The present study was undertaken to compare the effectiveness of NPWT and 3% hypertonic saline dressing in the management of diabetic foot ulcers.

Material and methods: This prospective comparative study was conducted in the Department of General Surgery, Employees' State Insurance Corporation (ESIC) Medical College and Hospital, Hyderabad, India, between January 2022 and December 2024. A total of 230 patients with Wagner grade 1 and 2 diabetic foot ulcers were included. Following surgical debridement and infection control measures, 115 patients were treated with NPWT, and 115 patients received 3% hypertonic saline dressings. Patients were followed for four weeks. Outcomes assessed included wound healing, duration of hospital stay, requirement for debridement, and amputation rate.

Results: Baseline demographic and clinical characteristics were comparable between the two groups. Mean wound dimensions in the NPWT group decreased from 8.59 ± 1.43 cm × 4.32 ± 1.25 cm to 3.20 ± 1.12 cm × 2.52 ± 1.32 cm, whereas in the hypertonic saline group they decreased from 7.98 ± 1.34 cm × 4.80 ± 1.85 cm to 5.25 ± 1.48 cm × 2.78 ± 1.89 cm (p < 0.05). The mean hospital stay was significantly shorter in the NPWT group (19.17 ± 2.3 vs. 35.12 ± 3.8 days). Healing rates were higher (91.3% vs. 78.3%), and amputation rates were lower (8.7% vs. 21.7%) in the NPWT group.

Conclusion: NPWT demonstrated superior wound healing outcomes compared with 3% hypertonic saline dressing in patients with diabetic foot ulcers. The therapy resulted in enhanced wound contraction, reduced hospitalization, and improved clinical outcomes. These findings support the use of NPWT as an effective treatment modality in the management of diabetic foot ulcers.

## Introduction

Diabetic foot ulcers represent one of the most serious and challenging complications of diabetes mellitus, contributing substantially to patient morbidity, mortality, and healthcare expenditure worldwide. The estimated lifetime risk of developing a diabetic foot ulcer ranges from 19% to 34%, with recurrence remaining common even after successful healing [1-3]. Diabetic foot ulcers frequently become complicated by infection, prolonged hospitalization, and lower-extremity amputation, resulting in significant functional impairment and reduced quality of life. Furthermore, mortality following major amputation remains alarmingly high, emphasizing the clinical and socioeconomic burden associated with this condition [4]. The increasing global prevalence of diabetes has led to a parallel rise in foot-related complications, particularly in low- and middle-income countries, where delayed presentation, limited healthcare resources, and restricted access to specialized wound care services often contribute to poorer clinical outcomes [2]. Consequently, the effective management of diabetic foot ulcers remains a major priority in contemporary surgical and diabetic care.

The pathogenesis of diabetic foot ulcers is multifactorial and involves a complex interaction between peripheral neuropathy, peripheral arterial disease, and impaired immune and reparative mechanisms resulting from chronic hyperglycemia [5-7]. Sensory neuropathy predisposes patients to repetitive unnoticed trauma, while motor neuropathy leads to structural foot deformities and abnormal pressure distribution, increasing the risk of tissue breakdown. Simultaneously, vascular insufficiency compromises tissue perfusion and oxygen delivery, thereby delaying wound healing and promoting chronic ulceration. Hyperglycemia further impairs leukocyte function, collagen synthesis, and cellular regeneration, increasing susceptibility to infection and reducing healing potential. Therefore, successful treatment requires a comprehensive approach encompassing glycemic control, vascular assessment, infection management, pressure offloading, and appropriate wound bed preparation [8,9]. Among advanced wound-care modalities, negative pressure wound therapy (NPWT) has gained widespread acceptance because of its ability to promote wound contraction, stimulate granulation tissue formation, enhance angiogenesis, reduce interstitial edema, and improve the wound-healing environment through controlled subatmospheric pressure [10-14]. In contrast, hypertonic saline dressing facilitates wound healing primarily through osmotic action, promoting autolytic debridement and reducing microbial burden [15]. Although both treatment modalities are widely employed in clinical practice, evidence comparing their effectiveness in specific institutional and regional settings remains limited. Therefore, the present study was undertaken to evaluate and compare the clinical effectiveness of NPWT and 3% hypertonic saline dressings in the management of diabetic foot ulcers.

## Materials and methods

This prospective comparative study was conducted in the Department of General Surgery, Employees' State Insurance Corporation (ESIC) Medical College and Hospital, Hyderabad, India, between January 2022 and December 2024, following approval from the Institutional Ethics Committee of the institution (approval number: ESICMC/SNR/IEC-S0230/04-2023). Adult patients presenting to the general surgery outpatient and inpatient departments with diabetic foot ulcers were screened for eligibility and recruited consecutively after obtaining written informed consent. A total of 230 patients diagnosed with diabetic foot ulcers were enrolled in the study.

Patients aged 18 years and above with Wagner grade 1-2 diabetic foot ulcers, classified according to the Wagner ulcer grading system [10], were included. Individuals with untreated osteomyelitis, critical limb ischemia not suitable for negative pressure wound therapy, suspected malignant ulcers, or those unwilling to participate were excluded. All enrolled patients underwent detailed clinical evaluation, including documentation of ulcer duration, ulcer dimensions, Wagner grading, and assessment of peripheral pulses. Vascular status was assessed using Ankle-Brachial Index (ABI) measurement where indicated [11]. Baseline laboratory investigations included hemoglobin, total leukocyte count, fasting and postprandial blood glucose levels, glycated hemoglobin (HbA1c), serum albumin, and renal function tests.

Following adequate wound debridement and infection control, patients were allocated into two treatment groups comprising 115 patients each. One group received NPWT using a standardized vacuum-assisted closure system delivering continuous negative pressure of approximately 125 mmHg, with dressing changes performed every 48-72 hours. The other group received sterile gauze dressings soaked in 3% hypertonic saline solution, which were changed daily under aseptic precautions according to the study protocol. Patients in both groups received standard diabetic care, including glycemic optimization, antibiotic therapy when indicated, pressure off-loading, and nutritional support. Patients were followed for four weeks, and outcomes assessed included reduction in wound dimensions, duration of hospital stay, requirement for debridement, wound healing, and amputation rate.

Statistical analysis

Data were entered into Microsoft Excel (Microsoft Corp., Redmond, WA, USA) and analyzed using IBM SPSS Statistics for Windows, Version 26.0 (IBM Corp., Armonk, New York, USA). Continuous variables were expressed as mean ± standard deviation and compared using Student's t-test. Categorical variables were expressed as frequencies and percentages and compared using the chi-square test. A p-value of less than 0.05 was considered statistically significant.

## Results

A total of 230 adult patients with diabetic foot ulcers were included in the study, with 115 patients managed using NPWT and 115 patients treated with 3% hypertonic saline dressings. Baseline demographic and clinical characteristics, including age, sex, duration of diabetes, ulcer size, Wagner grade, and ulcer location, were comparable between the two groups, with no statistically significant differences (p>0.05) (Table 1)

**Table 1:** Baseline demographic and clinical characteristics of the study population Data are presented as mean ± standard deviation (SD) or number (%). Student's t-test was used for continuous variables and the chi-square (χ²) test for categorical variables. p < 0.05 was considered statistically significant. NPWT: negative pressure wound therapy

Parameters	NPWT (n=115)	Hypertonic Saline (n=115)	Test Statistic	p-value
Age (years)	50.65 ± 8.31	49.98 ± 8.10	t = 1.38	0.170
Gender			χ² = 0.04	0.841
Male	71 (61.7%)	78 (67.8%)		
Female	44 (38.3%)	37 (32.2%)		
Wound size			χ² = 1.65	0.199
<5 cm	59 (51.3%)	63 (54.8%)		
≥5 cm	56 (48.7%)	52 (45.2%)		
Wagner grade			χ² = 0.01	0.929
Grade 1	25 (21.7%)	30 (26.1%)		
Grade 2	90 (78.3%)	85 (73.9%)		
Duration of diabetes (years)	11.96 ± 4.30	12.52 ± 3.90	t = 0.68	0.497

Following treatment, patients managed with NPWT demonstrated significantly greater wound healing compared with those treated with 3% hypertonic saline dressing. Mean wound dimensions decreased from 8.59 ± 1.43 cm × 4.32 ± 1.25 cm to 3.20 ± 1.12 cm × 2.52 ± 1.32 cm in the NPWT group, whereas wounds in the hypertonic saline group decreased from 7.98 ± 1.34 cm × 4.80 ± 1.85 cm to 5.25 ± 1.48 cm × 2.78 ± 1.89 cm. The reduction in wound size was significantly greater in the NPWT group (p<0.05). The mean duration of hospital stay was significantly shorter in patients treated with NPWT compared with the hypertonic saline group (19.17 ± 2.3 days vs. 35.12 ± 3.8 days). In addition, the NPWT group required fewer debridement procedures and demonstrated higher healing rates (91.3% vs. 78.3%) with lower amputation rates (8.7% vs. 21.7%). These outcome measures are summarized (Table 2).

**Table 2:** Comparison of wound parameters before and after treatment Data are presented as mean ± standard deviation (SD) or number (%). Student's t-test was used for continuous variables and the chi-square test for categorical variables. p < 0.05 was considered statistically significant. NPWT: negative pressure wound therapy

Parameters	NPWT (n=115)	Hypertonic Saline (n=115)	Test Statistic	p-value
Pre-treatment length (cm)	8.59 ± 1.43	7.98 ± 1.34	t = 1.00	0.318
Pre-treatment width (cm)	4.32 ± 1.25	4.80 ± 1.85	t = 1.29	0.198
Positive culture before treatment	35 (30.4%)	32 (27.8%)	χ² = 0.39	0.534
Post-treatment length (cm)	3.20 ± 1.12	5.25 ± 1.48	t = 2.53	0.012
Post-treatment width (cm)	2.52 ± 1.32	2.78 ± 1.89	t = 2.60	0.01
Positive culture after treatment	14 (12.2%)	17 (14.8%)	χ² = 0.04	0.84
Hospital stay (days)	19.17 ± 2.3	35.12 ± 3.8	t = 38.51	<0.001

**Figure 1:**
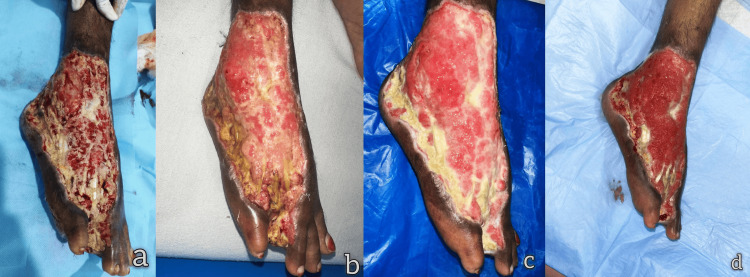
Clinical photographs demonstrating progressive wound healing in a diabetic foot ulcer during vacuum-assisted closure (VAC) therapy (a) Day 0 (baseline), showing a large wound with extensive slough and exposed tissue; (b) Day 6, showing reduction in slough and early granulation tissue formation; (c) Day 12, demonstrating further granulation and wound bed improvement; (d) Day 21, showing substantial wound contraction and a healthy granulating wound bed.

**Figure 2:**
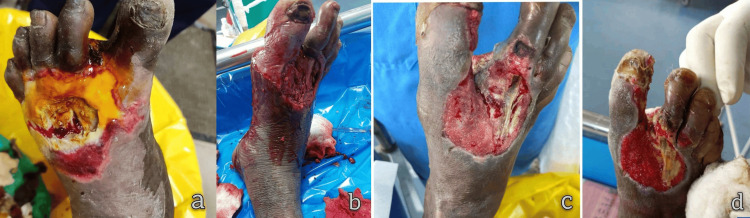
Clinical photographs demonstrating progressive wound healing in a diabetic foot ulcer during 3% hypertonic saline treatment (a) Day 0 (baseline), showing a wound with extensive slough; (b) Day 0, after debridement; (c) Day 9, showing further granulation and wound bed improvement; (d) Day 24, showing substantial wound contraction and a healthy granulating wound bed.

Patients treated with NPWT demonstrated a greater reduction in wound dimensions and improved wound bed preparation compared with those treated with hypertonic saline dressing. Representative clinical photographs illustrating wound healing progression following NPWT and hypertonic saline dressing are shown in Figures 1-2, respectively.

## Discussion

Diabetic foot ulceration develops as a consequence of complex metabolic, neurological, vascular, and immunological abnormalities associated with diabetes mellitus [1-3]. Chronic hyperglycemia leads to progressive damage of peripheral nerves and blood vessels, resulting in impaired wound healing and increased susceptibility to infection. The interaction of these pathological processes predisposes patients to chronic ulceration, recurrent hospitalization, and lower-extremity amputation. Consequently, diabetic foot ulcers remain a major cause of morbidity and healthcare expenditure worldwide [4].

The findings of the present prospective comparative study demonstrate that NPWT provides a significant therapeutic advantage over 3% hypertonic saline dressing in the management of diabetic foot ulcers. Patients treated with NPWT exhibited accelerated wound contraction, earlier and more robust granulation tissue formation, and reduced requirement for secondary surgical procedures. These improvements translated into superior limb salvage outcomes, particularly among individuals presenting with larger and more complex ulcers. The magnitude of benefit observed reinforces the growing body of evidence supporting the integration of NPWT into structured diabetic foot management protocols. The biological plausibility underlying these results is well established. NPWT exerts its therapeutic effects through macro-deformation and micro-deformation mechanisms. Controlled sub-atmospheric pressure approximates wound edges, thereby reducing wound volume, while simultaneously inducing mechanical stress at the cellular level that stimulates fibroblast proliferation, angiogenesis, and extracellular matrix deposition. Continuous removal of interstitial fluid decreases tissue edema and improves capillary perfusion, enhancing oxygen and nutrient delivery to the wound bed. These synergistic mechanisms collectively promote accelerated tissue regeneration and epithelialization [12-14]. In contrast, hypertonic saline dressing primarily facilitates osmotic debridement and reduction of microbial burden, without providing mechanical stimulation or perfusion enhancement, which may account for the comparatively slower healing trajectory observed in this study [15].

The superiority of NPWT demonstrated in the present study is consistent with previously published randomized controlled trials and systematic reviews. Multiple high-quality investigations have reported improved healing rates, enhanced granulation tissue formation, and reduced time to wound closure with NPWT compared to conventional dressing methods. A summary of key comparative studies is presented in Table 3, illustrating the consistency of evidence favoring NPWT across diverse clinical settings and study designs.

**Table 3:** Literature review of studies on negative pressure wound therapy (NPWT), conventional wound care, and hypertonic saline in diabetic foot ulcers

Author	Year	Study Design	Sample size	Comparison	Principal outcome
Armstrong & Lavery [16]	2005	Randomized controlled trial (RCT)	162	Negative pressure wound therapy (NPWT) vs. Standard moist dressing	Higher healing rates and faster wound closure in the NPWT group
Blume et al. [17]	2008	Multicenter RCT	342	NPWT vs. Advanced moist therapy	Significantly greater complete wound closure with NPWT
Dumville et al. [18]	2018	Cochrane systematic review	11	NPWT vs. Conventional Therapy	Moderate-quality evidence supporting improved healing with NPWT
Liu et al. [19]	2017	Meta-analysis	9	NPWT vs. Conventional dressings	Reduced healing time and improved closure rates
Ubbink et al. [20]	2008	Systematic review	Multiple trials	NPWT application vs. Conventional therapy	NPWT may improve wound healing outcomes, although higher-quality studies are required
Driver et al. [21]	2010	Prospective RCT	162	NPWT vs. Moist wound therapy	Faster granulation tissue formation with NPWT
Vikatmaa et al. [22]	2008	Systematic review	Multiple trials	NPWT vs. Conventional therapy	NPWT is associated with reduced wound size and better outcomes

Consistent with these studies, the present analysis also demonstrated a size-dependent therapeutic benefit, wherein larger ulcers derived proportionately greater advantage from NPWT [22]. Larger wounds are typically characterized by increased exudate production, interstitial edema, and impaired perfusion; thus, the fluid evacuation and mechanical stimulation provided by NPWT may exert a more pronounced clinical effect in this subgroup. This observation is particularly relevant for surgical practice, as larger ulcers are often associated with higher amputation risk.

Another clinically meaningful finding of this study was the reduced requirement for secondary surgical interventions in the NPWT cohort. Rapid optimization of the wound bed may reduce the necessity for repeated debridement or additional operative procedures. Although long-term amputation rates were not specifically evaluated beyond the study period, improved short-term limb salvage rates suggest potential downstream benefits. From a healthcare systems perspective, earlier wound stabilization may reduce hospitalization duration and overall treatment burden, though formal cost-effectiveness analysis was beyond the scope of this study [23]. Despite its advantages, NPWT requires specialized equipment, technical expertise, and higher upfront costs, which may limit universal applicability. Future research should focus on developing low-cost NPWT systems, reusable components, and simplified treatment protocols to improve affordability and accessibility, particularly in resource-limited settings. Furthermore, larger multicenter studies with longer follow-up are warranted to evaluate long-term wound recurrence, limb salvage, quality of life, and the overall cost-effectiveness of NPWT in the management of diabetic foot ulcers. Hypertonic saline dressing remains an inexpensive and accessible alternative, particularly in resource-constrained environments. Therefore, wound care decisions should be individualized, considering ulcer characteristics, vascular status, infection severity, patient comorbidities, and institutional resources.

The present study was conducted at a single tertiary care center, which may limit the generalizability of the findings. The follow-up period was limited to four weeks, and, therefore, long-term wound recurrence, functional outcomes, and quality of life measures could not be assessed. Additionally, formal cost-effectiveness analysis of NPWT was not performed. Further multicenter studies with longer follow-up durations are required to validate these findings.

## Conclusions

This prospective comparative study demonstrates that NPWT provides a significant therapeutic advantage over 3% hypertonic saline dressing in the management of diabetic foot ulcers. NPWT was associated with accelerated wound contraction, earlier granulation tissue formation, reduced need for secondary surgical procedures, and improved limb salvage rates, particularly in larger ulcers. These outcomes highlight the biological and mechanical benefits of controlled subatmospheric pressure in optimizing the wound microenvironment and promoting tissue regeneration. Although hypertonic saline dressing remains a cost-effective and accessible modality, its comparatively slower rate of wound contraction suggests that it may be better suited for smaller or less complex ulcers, especially in resource-constrained settings. Clinical decision-making should therefore incorporate ulcer size, severity, vascular status, and institutional resource availability when selecting an appropriate wound care strategy.
